# PERMANOVA-S: association test for microbial community composition that accommodates confounders and multiple distances

**DOI:** 10.1093/bioinformatics/btw311

**Published:** 2016-05-19

**Authors:** Zheng-Zheng Tang, Guanhua Chen, Alexander V. Alekseyenko

**Affiliations:** ^1^Department of Biostatistics, Vanderbilt University School of Medicine, Nashville, TN 37203, USA; ^2^Biomedical Informatics Center,; ^3^Department of Public Health Sciences and; ^4^Department of Oral Health Sciences, Medical University of South Carolina, Charleston, SC 29403, USA

## Abstract

**Motivation:** Recent advances in sequencing technology have made it possible to obtain high-throughput data on the composition of microbial communities and to study the effects of dysbiosis on the human host. Analysis of pairwise intersample distances quantifies the association between the microbiome diversity and covariates of interest (e.g. environmental factors, clinical outcomes, treatment groups). In the design of these analyses, multiple choices for distance metrics are available. Most distance-based methods, however, use a single distance and are underpowered if the distance is poorly chosen. In addition, distance-based tests cannot flexibly handle confounding variables, which can result in excessive false-positive findings.

**Results:** We derive presence-weighted UniFrac to complement the existing UniFrac distances for more powerful detection of the variation in species richness. We develop PERMANOVA-S, a new distance-based method that tests the association of microbiome composition with any covariates of interest. PERMANOVA-S improves the commonly-used Permutation Multivariate Analysis of Variance (PERMANOVA) test by allowing flexible confounder adjustments and ensembling multiple distances. We conducted extensive simulation studies to evaluate the performance of different distances under various patterns of association. Our simulation studies demonstrate that the power of the test relies on how well the selected distance captures the nature of the association. The PERMANOVA-S unified test combines multiple distances and achieves good power regardless of the patterns of the underlying association. We demonstrate the usefulness of our approach by reanalyzing several real microbiome datasets.

**Availability and Implementation:** miProfile software is freely available at https://medschool.vanderbilt.edu/tang-lab/software/miProfile.

**Contact:** z.tang@vanderbilt.edu or g.chen@vanderbilt.edu

**Supplementary information: **Supplementary data are available at *Bioinformatics* online.

## 1 Introduction

Biological and empirical evidences suggest that a diverse microbial community plays an important role in human health (Alekseyenko *et al.*, 2013; Redel *et al.*, 2013; Segal *et al.*, 2013). Understanding the composition of the microbial community provides insight into the functions of bacteria and their effects on the human host. Sequencing technology has made it possible to capture high-throughput data on the microbiome composition in human specimens. A common unit of analysis for sequencing-based microbiome identification studies is the operational taxonomic unit (OTU). OTUs represent the conceptualization of species and are typically constructed by clustering sequences at a certain similarity threshold (e.g. 97%) (Caporaso *et al.*, 2010; Schloss *et al.*, 2009). Comparison of OTU profiles with respect to differential clinical outcomes or conditions lends important knowledge towards understanding the underlying disease mechanisms and the effects of dysbiosis (Alekseyenko *et al.*, 2013; Cho *et al.*, 2012).

Testing associations of the microbiome composition with covariates of interest is challenging because the sample size is typically modest and the number of OTUs is large. Distance-based omnibus tests are popular, as these tests successfully address the challenges by partitioning the distance matrix among sources of variation and evaluating the statistical significance by permutation (Anderson, 2001a; McArdle and Anderson, 2001). Specifically, when testing for the community difference between groups, the distance-based method contrasts the pairwise distance within and between groups. This statistic is an analogue to Fisher’s *F*-ratio.

The difference between two microbial composition profiles follows various patterns. The difference may exist in one or several clusters of the species on the phylogenetic tree (lineages) or random sets of species. The difference may be driven by the change of species richness, evenness or a combination of the two. Richness and evenness are the two main factors that describe species diversity in microbial communities. Each of these measures addresses a different aspect of community ecology; thus, by considering them together, a much more insightful picture of the community structure is provided. Increased richness and evenness are often associated with more stable and longer established ecosystems (Legendre and Legendre, 2012; Relman, 2012). Such ecosystems tend to be resistant to environmental pressures, such as diet, antibiotic use and pathogen invasion (Cox *et al.*, 2013; Shade *et al.*, 2012).

Over two dozen distance measures are available in open source packages, like vegan (Oksanen *et al.*, 2015), ade4 (Dray and Dufour, 2007) and phyloseq (McMurdie and Holmes, 2013), to quantify the variation in composition between microbiome samples (beta diversity). The unweighted and weighted UniFrac are two commonly-used distances constructed on the phylogenetic tree (Lozupone and Knight, 2005; Lozupone *et al.*, 2007). These distances are efficient in detecting differential species in lineages of the tree. The unweighted UniFrac uses the difference of the species presence–absence status between two samples to determine the inclusion of a particular tree branch. The weighted UniFrac uses the difference of the species relative abundance (proportion) between two samples to weight the branch. As an extension of the weighted UniFrac, the generalized UniFrac introduces a parameter to attenuate the contribution from high abundant lineages (Chen *et al.*, 2012). Bray-Curtis and Jaccard distances are two popular dissimilarity measures that do not utilize the phylogenetic tree. They tend to be efficient in detecting association on arbitrary species rather than in lineages. Bray-Curtis distance is defined as the difference of the abundance divided by the total abundance contributed by both samples. Jaccard distance is defined as the number of unique species present in either sample, divided by the number of species present in any of the two samples.

Although distance-based methods provide powerful tools to discover associations between the microbiome composition and covariates, they suffer from two main limitations. First, existing distance-based tests cannot flexibly handle confounders, which are defined as variables correlated with both the covariates of interest and the microbiome composition. Extending these methods to accommodate more-sophisticated outcomes and study designs (Lin and Zeng, 2009; Lin *et al.*, 2013) in the presence of confounders is challenging (Zhao *et al.*, 2015). Treating confounders as covariates or omitting the confounders in the distance-based test distorts the true association. Hence, the ability to properly model and adjust for confounders becomes critically important. Second, existing distance-based tests cannot simultaneously incorporate multiple distances. As the underlying biology is unknown a priori, the selected distance is most likely suboptimal and may not yield good statistical power. Searching multiple distances and reporting the one that produces the smallest *P*-value is too liberal and yields excessive false discoveries, unless adjustments are made for multiple comparisons. On the other hand, adjustments for multiple comparisons may result in poor power when an excessive number of distances is tested. Hence, it is highly desirable to have a unified test that combines multiple distances.

In this article, we derive presence-weighted UniFrac to complement the existing UniFrac distances for more powerful detection of variation in species richness. The presence-weighted UniFrac distances achieve adequate power in a wide range of scenarios. We develop PERMANOVA-S, a new distance-based method to test the association of microbial communities with any covariates of interest. PERMANOVA-S improves the commonly-used Permutation Multivariate Analysis of Variance (PERMANOVA) test by allowing flexible confounder adjustments and ensembling multiple distances. We conducted extensive simulations to evaluate the performance of different distances under various patterns of association. Our studies demonstrate that the power of the test relies on how well the selected distance captures the nature of the association. The PERMANOVA-S unified test combines multiple distances and achieves good power regardless of the patterns in the underlying association. Application to real microbiome datasets demonstrates the usefulness of the proposed methods. The relevant software miProfile incorporating the new development is freely available.

## 2 Methods

The PERMANOVA is the most commonly applied distance-based method to test the association of microbial composition with covariates of interest. The test statistics directly use the distance matrix to partition the diversity among sources of variation. This test is especially suitable for the analysis of composition data from ecology studies with a small sample size and a large number of features.

Suppose we generate the microbiome profile **Y** with *m* OTUs and collect the covariates **X** for *n* samples. We compute a distance matrix **D** that quantifies the dissimilarity between samples based on **Y** and potentially the phylogenetic structure of the OTUs. The pseudo-*F* test statistic is defined as

$$F=\frac{\text{tr}(HGH)}{\text{tr}((I-H)G(I-H))},$$

where tr(·) is the trace operator on a matrix, $H=X{\left(X^{\text{T}}X\right)}^{-1}X^{\text{T}}$, and $G=-\frac{1}{2}(I-\frac{11^{\text{T}}}{n})D^{2}(I-\frac{11^{\text{T}}}{n})$, where **I** is an *n *×* n* identity matrix, **1** is an n×1 vector consisting of ones and **D**^2^ is the element-wise square of **D**. The significance of the pseudo-*F* statistic is evaluated by simulating the null distribution from permutations.

### 2.1 Abundance and presence–absence distances

We categorize all distances into abundance distances and presence–absence distances according to the forms of the microbiome data they use: abundance distances are based on the abundance data (counts or proportions) of the species, and the presence–absence distances are based on the presence–absence data (binary indicator for the presence) of the species.

Among the distances that do not utilize the tree information, Bray-Curtis distance is an abundance distance and Jaccard distance is a presence–absence distance. Let *p_ji_* and *p_ki_* denote the abundance of OTU *i* in samples *j* and *k*, respectively. Bray-Curtis distance between sample *j* and *k* is defined as

$$D_{\text{BC}}=\frac{\sum_{i=1}^{m}\left|p_{ji}-p_{ki}\right|}{\sum_{i=1}^{m}\left(p_{ji}+p_{ki}\right)}.$$

Let $n_{jk}^{01}$ and $n_{jk}^{11}$ denote the count of the species present in only one sample or both samples, respectively. Jaccard distance is defined as

$$D_{\text{J}}=\frac{n_{jk}^{01}}{n_{jk}^{01}+n_{jk}^{11}}.$$

After converting the abundance data to the presence–absence data (code the present OTU to 1 and the absent OTU to 0), **D**_BC_ becomes $n_{jk}^{01}/(n_{jk}^{01}+2n_{jk}^{11})$. This expression is almost identical to **D**_J_ except for a factor of 2 multiplied to $n_{jk}^{11}$ in the denominator. This difference has ignorable effects in terms of discriminating samples, and we consider the presence–absence version of Bray-Curtis distance as equivalent to the Jaccard distance.

Among the tree-based distances, weighted UniFrac and generalized UniFrac are abundance distances, and unweighted UniFrac is a presence–absence distance. Suppose we have a phylogenetic tree with *L* branches associated with the OTUs. Let *b_l_* denote the length of branch *l*, and *p_jl_* and *p_kl_* denote the proportion of the OTUs descending from branch *l* for samples *j* and *k*, respectively. The weighted UniFrac and generalized UniFrac distances between samples *j* and *k* are defined as

$$\begin{array}{l} D_{\text{W}}=\frac{\sum_{l=1}^{L}b_{l}|p_{jl}-p_{kl}|}{\sum_{l=1}^{L}b_{l}(p_{jl}+p_{kl})}\;\;\text{and}\\ D_{\text{GW}}^{\left(\alpha \right)}=\frac{\sum_{l=1}^{L}b_{l}{\left(p_{jl}+p_{kl}\right)}^{\alpha }\left|\frac{p_{jl}-p_{kl}}{p_{jl}+p_{kl}}\right|}{\sum_{l=1}^{L}b_{l}{\left(p_{jl}+p_{kl}\right)}^{\alpha }}, \end{array}$$

respectively, where α takes a value between 0 and 1. The lower value corresponds to weaker contribution from the high abundant branches. The distance $D_{\text{GW}}^{\left(\alpha \right)}$ reduces to **D**_W_ when α = 1.

Let *n_jl_* and *n_kl_* denote the number of present OTUs descending from branch *l* for samples *j* and *k*, respectively. Unweighted UniFrac is defined as

$$D_{\text{UW}}=\frac{\sum_{l=1}^{L}b_{l}\left|I(n_{jl}>0)-I(n_{kl}>0)\right|}{\sum_{l=1}^{L}b_{l}},$$

where I(·) is the indicator function.

For generalized UniFrac, the corresponding version based on the presence–absence data can be expressed as

$$D_{\text{PW}}^{\left(\alpha \right)}=\frac{\sum_{l=1}^{L}b_{l}{\left(n_{jl}+n_{kl}\right)}^{\alpha }\left|\frac{n_{jl}-n_{kl}}{n_{jl}+n_{kl}}\right|}{\sum_{l=1}^{L}b_{l}{\left(n_{jl}+n_{kl}\right)}^{\alpha }}.$$

Distinct from $D_{\text{UW}},\;D_{\text{PW}}^{\left(\alpha \right)}$ uses the relative richness difference $\left|n_{jl}-n_{kl}|/(n_{jl}+n_{kl}\right)$ to weight the branch. By decreasing α in $D_{\text{PW}}^{\left(\alpha \right)}$, we attenuate the contribution from the high rich branches and make the dissimilarity measures more sensitive to the richness change on the moderately rich lineage. The distance $D_{\text{PW}}^{\left(\alpha \right)}$ is referred to as presence-weighted UniFrac.

### 2.2 PERMANOVA-S

We develop a new method, PERMANOVA-S, to flexibly accommodate confounders and multiple distances. We first explain the method for confounder adjustment in the presence of a single distance. Then, we tailor the permutation procedure to combine multiple distances.

#### 2.2.1 Confounder adjustment

In the presence of confounding variables Z, we permute residuals after regressing **X** on Z. We let γZ denote the linear predictor in the regression model.

If **X** is a continuous variable, we use standard linear regression to compute the residual and the Freedman–Lane permutation strategy (Freedman and Lane, 1983) to obtain the *P*-value.

1. Regress **X** on Z and obtain the maximum likelihood estimator (MLE) $\hat{\gamma }$ for γ, compute the residuals $R=X-\hat{\gamma }Z$ and construct the observed pseudo-*F* statistic based on R.
2. For each permutation, permute R to yield $R^{*}$, and replace **X** by $X^{*}=\hat{\gamma }Z+R^{*}$. Regress $X^{*}$ on Z and obtain the MLE ${\hat{\gamma }}^{*}$ for γ, compute the residuals $R^{**}=X^{*}-{\hat{\gamma }}^{*}Z$ and construct the permuted pseudo-*F* statistic based on $R^{**}$.
3. The final *P*-value is the proportion of the permuted pseudo-*F* statistics larger than the observed statistic.

If **X** is a binary variable, we use standard logistic regression to compute the residual and the parametric bootstrap (Davison and Hinkley, 1997) to obtain the *P*-value.

1. Regress **X** on Z and obtain the MLE estimate $\hat{\gamma }$ for γ, compute the residuals $R=X-\frac{\exp(\hat{\gamma }Z)}{1+\exp(\hat{\gamma }Z)}$ and construct the observed pseudo-*F* statistic based on R.
2. For each permutation, sample $X^{*}$ from Bernoulli distribution with probability parameter $\frac{\exp(\hat{\gamma }Z)}{1+\exp(\hat{\gamma }Z)}$. Regress $X^{*}$ on Z and obtain the MLE ${\hat{\gamma }}^{*}$ for γ, compute the residuals $R^{**}=X^{*}-\frac{\exp({\hat{\gamma }}^{*}Z)}{1+\exp({\hat{\gamma }}^{*}Z)}$ and construct the permuted pseudo-*F* statistic based on $R^{**}$.
3. The final *P*-value is the proportion of the permuted pseudo-*F* statistics larger than the observed statistic.

We have implicitly assumed that the covariate **X** is univariate and that the samples are unrelated. For repeated measures or paired-sample studies, we restrict the permutation within each stratum. For more complicated designs and data types, we need to seek suitable permutation strategies (Anderson, 2001b; Pesarin and Salmaso, 2010). A comprehensive discussion of the existing strategies is beyond the scope of this paper.

In order to control confounding effects, sequential *F*-test partitions the distance matrix first with respect to confounders and second with respect to covariates of interest by fitting linear models to distance matrices (Oksanen *et al.*, 2015). Our approach is conceptually different because we obtain residuals through regression of the covariates on confounders. Although the two approaches are equivalent under certain scenarios (e.g. continuous covariates, standard linear model), our approach is more flexible because suitable models can be employed according to the types of covariates and study designs.

#### 2.2.2 Ensembling of distances

Choosing the distance sensitive to the patterns of association yields high power in the association test. Instead of considering one distance at a time, we develop a multistage permutation strategy that enables us to ensemble multiple distances in a computationally efficient manner. When unifying multiple distances, we use the minimal *P*-value across distances as the test statistic. To obtain the unified *P*-value, we employ a permutation procedure to simulate the null distribution for the minimal *P*-value. The procedure does not depend on a large-sample approximation, so it can be used in a study with a small sample size. Suppose we consider *K* distances $D_{1},D_{2},\ldots ,D_{K}$. The testing procedure is described below

1. For each **D**_*k*_, compute the observed pseudo-*F* statistic *F_k_*.
2. Simultaneously generate *B* permuted pseudo-*F* statistics $F_{k}^{\left(1\right)},\ldots ,F_{k}^{\left(B\right)}$ for the *K* distances. We suggest starting the value of *B* from a small value (e.g. 500). As noted in the previous section, the selected permutation strategy should be based on the study design and the data types.
3. For each **D**_*k*_, obtain the *P*-value *p_k_* based on the *B* permutations. If $p_{\text{min}}=\min(p_{1},\ldots ,p_{K})<1/(\text{tol}\times B)$, increase *B* and repeat step 2. Otherwise, go to step 4. The pre-specified parameter *tol* takes a value between 0 and 1, with a smaller value corresponding to a more stringent accuracy requirement.
4. For each **D**_*k*_, calculate *B* permuted ‘*P*-values’ $p_{k}^{\left(1\right)},\ldots ,p_{k}^{\left(B\right)}$ as $p_{k}^{\left(b\right)}=(B-\text{rank}(F_{k}^{\left(b\right)}))/B$, where $\text{rank}(F_{k}^{\left(b\right)})$ is the rank of $F_{k}^{\left(b\right)}$ among *B* permuted statistics. This step can be efficiently completed by the quick sort algorithm.
5. For each permutation, obtain the minimal permuted ‘*P*-value’ across *K* distances: $p_{\text{min}}^{\left(b\right)}=\min(p_{1}^{\left(b\right)},\ldots ,p_{K}^{\left(b\right)})$.
6. The final *P*-value is the proportion of $p_{\text{min}}^{\left(1\right)},\ldots ,p_{\text{min}}^{\left(B\right)}$ that is smaller than *p*_min_.

To obtain the exact *P*-values for each permuted pseudo-*F* statistic in step 2, one needs to perform another layer of permutation, which is computationally infeasible. In our procedure, the permuted ‘*P*-values’ obtained in step 4 are based on the original round of permutation. The accuracy of these ‘*P*-values’ depends on the value of *B*. By adopting the multistage procedure in step 3, we subsequently find a *B* that makes these ‘*P*-values’ accurate enough to calculate the unified *P*-value. The existence of such *B* is because of the implicit fact that the unified *P*-value cannot be smaller than the minimal *P*-value across distances. In addition, the multistage procedure ensures that the value of *B* is not so large as to squander computation time.

### 2.3 Software: miProfile

We have developed a software program miProfile that incorporates the new development. miProfile implements all commonly-used abundance and presence–absence distances. Users can request any combination of these distances. miProfile produces the *P*-value for each of the requested distances and the unified *P*-value by combining all of the distances. The operating interface is simple and can directly input the .biom and .tre files. The OTU abundance table and the distance matrices can be generated as output and reused in subsequent runs. The permutation implemented in miProfile is optimized to achieve computational efficiency. It can finish one million permutations within 150 seconds on an IBM HS22 machine for 100 samples.

### 2.4 Simulation strategies

We performed extensive simulation studies to investigate the type I error of PERMANOVA-S and to compare the power of abundance distances, presence–absence distances and the PERMANOVA-S unified test in the detection of various association patterns. Following Chen *et al.* (2012), we generated 856 OTUs under the Dirichlet-multinomial (DM) model with proportion and dispersion parameters estimated from a real respiratory tract microbiome dataset (Charlson *et al.*, 2010). Then, we partitioned the 856 OTUs into 20 lineages via the Partitioning Around Medoids algorithm based on the patristic distance. We considered total sample sizes of 20 and 50 and split them into two groups. We assumed the sequencing depth was 1000 reads per sample. For all simulation studies, we considered six distances: weighted UniFrac (**D**_W_), Bray-Curtis (**D**_BC_), presence-weighted UniFrac with parameter 1 or 0 ($D_{\text{PW}}^{\left(1\right)},\;D_{\text{PW}}^{\left(0\right)}$), unweighted UniFrac (**D**_UW_) and Jaccard (**D**_J_). We performed the PERMANOVA-S unified test across these six distances. For comparison, we conducted the Bonferroni-adjusted test (Pmin) which uses the minimal *P*-value of the single-distance test and multiplies it by six.

For the type I error simulation, we considered scenarios with and without confounding effects. When no confounders exist, the sample is evenly distributed between the two groups. To simulate a confounding effect, we first generated a variable related to the microbial community. We then assigned the group label based on the value of that variable. Specifically, we simulated a variable Z from the normal distribution with the mean being the standardized abundance or richness of the most common lineage (19.7%), and we assigned the sample to group one with probability exp⁡(0.5Z)/(1+exp⁡(0.5Z)). We used 10 000 replicates to evaluate the type I error at a significance level of 0.05.

For the power simulation, we focused on evaluating the performance of different single-distance tests and the unified test. We considered the situation with no confounding effects. The unified test was provided using PERMANOVA-S to combine all six distances. We assumed that the differentiation between groups occurred in one of four possible OTU sets: a common lineage (19.7%), a rare lineage (0.9%), a random lineage, or random 40 OTUs. In addition, we designed three patterns of microbial community differentiation.

**Fig. 1.**
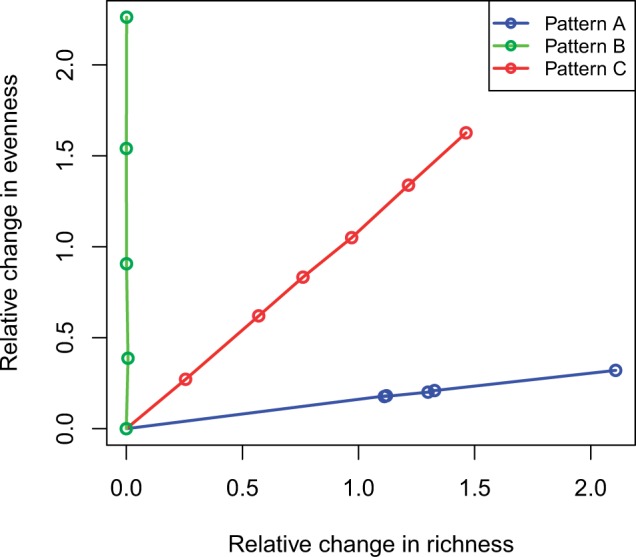
Relative change in richness (total number of present OTUs) and evenness (Shannon’s diversity index) between two groups for the three patterns of differentiation in a random lineage. Each dot represents the effect size we considered in the simulation studies. The plots for the common lineage, rare lineage and random 40 OTUs are similar and not shown
- For each OTU *i*, we increased the relative abundance by a factor of ${\left(\frac{1}{\sqrt{{\hat{p}}_{i}}}\right)}^{c}$ only at the presences in one group, where ${\hat{p}}_{i}$ is the average observed abundance in the other group. Pattern B dominantly changes evenness between groups without fluctuating richness (green curve in Fig. 1).
- For each OTU *i*, we increased the proportion parameters *p_i_* in DM distribution by a factor of ${\left(\frac{1}{\sqrt{p_{i}}}\right)}^{c}$ for one group. Pattern C changes richness and evenness at a balanced rate (red curve in Fig. 1).

- For each OTU *i*, we randomly changed absence (count of 0) to presence with count of 1 in one group, such that the resulting percentage of presences (positive counts) increased by a factor of *c*, where *c* controls the degree of differentiation. Pattern A dominantly changes richness between groups, while minimally fluctuating evenness (blue curve in Fig. 1).

For all patterns, we renormalized the relative abundance, such that the total proportion is equal to one. In all 12 possible scenarios from the combination of four OTU sets and three patterns of differentiation, we varied the degrees of the signal *c* and generated the power curves based on 2000 replicates at a significance level of 0.05.

## 3 Results

### 3.1 Simulation results

The empirical type I errors of PERMANOVA-S are shown in Table 1. When confounders exist, the test without confounder adjustment produces inflated type I error. The inflation occurs mostly on abundance distances (**D**_W_ and **D**_BC_) when the confounder is correlated with abundance and on presence–absence distances ($D_{\text{PW}}^{\left(1\right)},\;D_{\text{PW}}^{\left(0\right)},\;D_{\text{UW}}$ and **D**_J_) when the confounder is correlated with richness. The Bonferroni-adjusted test (Pmin) tends to be conservative.

**Table 1.** Empirical type I errors for PERMANOVA-S

	*n*	**D**_W_	**D**_BC_	$D_{\text{PW}}^{\left(1\right)}$	$D_{\text{PW}}^{\left(0\right)}$	**D**_UW_	**D**_J_	Pmin	Unified
No confounder									

	20	0.049	0.046	0.051	0.049	0.050	0.049	0.032	0.048
	50	0.048	0.051	0.050	0.044	0.042	0.047	0.035	0.050

Confounder *Z* correlated with abundance									

Adjust for Z	20	0.049	0.047	0.052	0.048	0.050	0.050	0.037	0.053
	50	0.051	0.048	0.052	0.051	0.050	0.052	0.035	0.052
Not adjust for Z	20	0.10	0.065	0.055	0.048	0.048	0.050	0.051	0.072
	50	0.20	0.10	0.054	0.052	0.053	0.054	0.094	0.13

Confounder *Z* correlated with richness									

Adjust for Z	20	0.053	0.053	0.051	0.051	0.051	0.052	0.036	0.053
	50	0.047	0.047	0.049	0.052	0.050	0.054	0.035	0.051
Not adjust for Z	20	0.055	0.051	0.074	0.054	0.052	0.056	0.044	0.061
	50	0.060	0.052	0.11	0.062	0.058	0.061	0.054	0.077

**Fig. 2.**
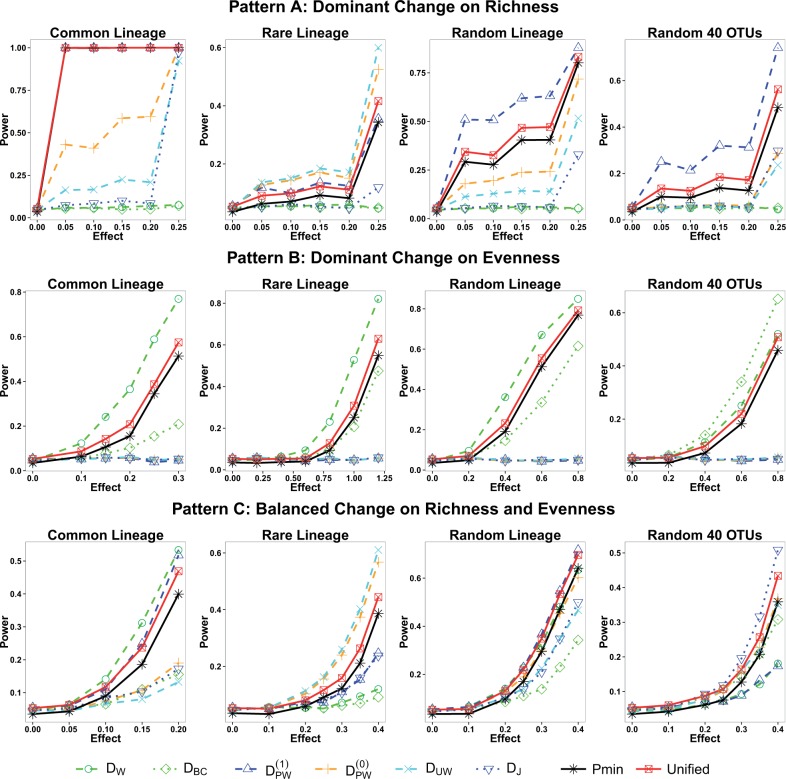
Power of abundance distances, presence–absence distances, Bonferroni-adjusted test and PERMANOVA-S unified test under various differentiation patterns. Each curve is created by varying the degree of differentiation between two groups, with 10 samples per group

Figure 2 shows the power results for three differentiation patterns when the sample size is 20 (see Supplementary Fig. S1 for results with a sample size of 50). Under pattern A, where the change of richness dominates the change of evenness (upper panel of Fig. 2), the presence–absence distances ($D_{\text{PW}}^{\left(1\right)},\;D_{\text{PW}}^{\left(0\right)},\;D_{\text{UW}},\;D_{\text{J}}$) are dramatically more powerful than the abundance distances ($D_{\text{W}},\;D_{\text{BC}}$) regardless of where differentiation appears. If the signal is on the common lineage, $D_{\text{PW}}^{\left(1\right)}$ (overlapping with the curve for the unified test) is more powerful than the other distances because it up-weights the high rich branches that the common lineage harbors. If the signal is on the rare lineage, **D**_UW_ produces the most powerful test, and $D_{\text{PW}}^{\left(0\right)}$ yields similar power as **D**_UW_. $D_{\text{PW}}^{\left(1\right)}$ is more powerful than the other distances if the signal is in a random lineage or on random OTUs.

Under pattern B, where the change of evenness dominates the change of richness (middle panel of Fig. 2), the use of the abundance distances is substantially more powerful than the use of the presence–absence distances, no matter where the differentiation occurs. **D**_W_ produces the most powerful test if the differentiation occurs in a lineage, and **D**_BC_ produces the most powerful test if the differentiation occurs on random OTUs.

Under pattern C, where the richness and evenness change at a balanced rate (lower panel of Fig. 2), abundance distances and the corresponding presence–absence distances produce more similar power than in patterns A and B. If differentiation occurs on the common or random lineage, the top two distances are abundance distance **D**_W_ and the corresponding presence–absence distance $D_{\text{PW}}^{\left(1\right)}$. If differentiation occurs on the rare lineage, the top two distances are **D**_UW_ and $D_{\text{PW}}^{\left(0\right)}$, as in pattern A. If differentiation occurs on random OTUs, **D**_J_ yields the top power.

The simulation study shows that the power for different distances can vary a lot across association patterns, and the proper choice of distance is essential for conducting well-powered association studies. In practice, the nature of the association usually appears as a mixture of these studied patterns, which makes the choice of the best distance an impossible mission. The Bonferroni-adjusted test is inevitably less powerful than the unified test because it ignores the correlation of the tests using different distances. The power loss of the Bonferroni-adjusted test will become larger if more distances are considered. Our simulation demonstrates that ensembling the abundance distances and presence–absence distances using the PERMANOVA-S unified test yields good discovery power regardless of the underlying association pattern.

### 3.2 Cutaneous microbiome in psoriasis

Psoriasis is a common chronic inflammatory disease of the skin. Alekseyenko *et al.* (2013) investigated the community differentiation of the cutaneous microbiota in psoriasis. A total of 51 patients with psoriasis were studied by swabbing the lesion and a contralateral sample of normal tissue from each subject. The microbial OTUs were constructed through the QIIME pipeline.

**Fig. 3.**
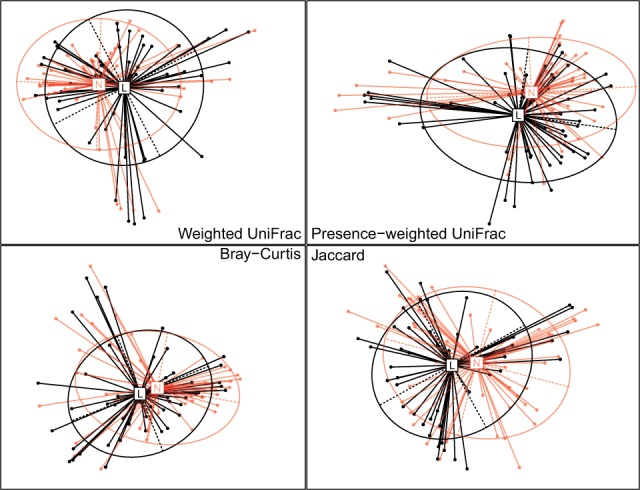
Comparison of abundance distances and presence–absence distances for discriminating lesion (L) and normal (N) samples. Principle component analysis is performed on the four distance matrices. The samples are plotted on the first two principle components. The ellipse center indicates groups means, and the height and width represent the variances on the two directions

After removing samples with read depth less than 1000 and discarding OTUs with only one read, we generated an OTU table of 50 complete pairs and 13 429 OTUs. We compared the microbial community composition between the lesion and normal samples from patients with psoriasis using PERMANOVA-S (one million within-subject permutations) and the six distances in the simulation studies. All of the distances give statistically significant results, which indicates that both the richness and evenness of the microbial community are different between the lesion and normal samples. However, the presence–absence distances ($D_{\text{PW}}^{\left(1\right)}$ *P*-value = $1.1\times 10^{-5}$; $D_{\text{PW}}^{\left(0\right)}$ *P*-value = $1.4\times 10^{-5}$; **D**_UW_ *P*-value = $1.6\times 10^{-5}$; **D**_J_ *P*-value = $1.3\times 10^{-5}$) produce stronger evidence of differentiation than the abundance distances (**D**_W_ *P*-value = $1.2\times 10^{-3}$; **D**_BC_ *P*-value = $4.7\times 10^{-4}$). The unified *P*-value is $3.1\times 10^{-5}$, which is close to the *P*-values of the presence–absence distances. We performed the principle coordinate analysis using the distance matrices $D_{\text{W}},\;D_{\text{PW}}^{\left(1\right)},\;D_{\text{BC}}$ and **D**_J_. We plotted the samples on the first two principle components (Fig. 3). The distances $D_{\text{PW}}^{\left(1\right)}$ and **D**_J_ separate the samples better than the distances **D**_W_ and **D**_BC_.

To identify the differential OTUs, we performed the Wilcoxon signed-rank test for paired samples based on the abundance data, and we performed the McNemar’s test based on the presence–absence data. We identified 10 OTUs from either of the tests at a significance level of 0.001 (Supplementary Table S1). The three related phyla were associated with psoriasis status and were employed to characterize and classify skin microbiota (Alekseyenko *et al.*, 2013). It is interesting to see that most of these OTUs have a significant *P*-value in only one test. If the association is mainly driven by the differential abundance, the Wilcoxon test yields more significant results than the McNemar’s test; if the association is mainly driven by the differential proportion of the presence, then the McNemar’s test produces more significant results (Supplementary Fig. S2).

This example demonstrates that the abundance data and the presence–absence data represent different views of the microbial community and the PERMANOVA-S unified test is a very powerful tool to identify the overall associations by combining the abundance and presence–absence distances.

### 3.3 Microbiome in subtherapeutic antibiotics and adiposity

Another study mimicked the common farm practice of administering low-dose antibiotics to promote animal growth. The laboratory model investigated an increase in adiposity after administration of low-dose antibiotic therapy to young mice and evaluated changes in body fat and composition of the gut microbiome (Cho *et al.*, 2012). Female mice were given four types of antibiotics or no antibiotics (control), with 10 samples in each group. The mass and percentage of body fat were measured. The microbes in the cecal contents of these animals were analyzed, and the OTUs were constructed through the QIIME pipeline. The study found that administration of low-dose antibiotic therapy increased adiposity in young mice and substantially changed the microbiome composition.

We deleted samples with low read depth and removed OTUs with only one read. In the final dataset, we had 48 samples and 2877 OTUs. We tested whether there is an association between gut microbes and body fat. We performed the unified test across the six distances in the simulation studies. First, we did not adjust for any potential confounders. The unified *P*-values produced marginally significant association with *P*-values of 0.058 and 0.069 for mass and percentage body fat, respectively. Among the six distances, the Jaccard distance produced the most significant *P*-values (0.019 for mass body fat and 0.025 for percentage body fat). We then adjusted for antibiotic treatment and repeated the analysis. The unified *P*-values became 0.19 and 0.35, which are less significant than the *P*-values of the tests without confounder adjustment. All of the *P*-values are listed in the Supplementary Table S2. Our analysis indicates that antibiotic treatment is a potential confounder when linking body fat to the gut microbiome composition. This example demonstrates the importance of confounder adjustment in the association analysis of microbiome composition.

## 4 Discussion

We derive the presence-weighted UniFrac based on the presence–absence data of the microbial species. Such distances are sensitive to variation of richness. We develop PERMANOVA-S as an improved version of PERMANOVA in order to facilitate the association study of microbiome composition. PERMANOVA-S is superior to PERMANOVA because it flexibly accommodates confounders and multiple distances. We investigated the type I error and power of PERMANOVA-S under different patterns of association by fluctuating the richness and evenness on various OTU sets. Our results show that PERMANOVA-S properly controls type I error, and the unified test is more robust than the single-distance test.

A method based on the maximum of the pseudo-*F* statistics (maxF) has been proposed to combine different distances (Chen *et al.*, 2012). The validity of the maxF test relies on the assumption that the reference distributions of the pseudo-*F* statistics are the same under different distances. PERMANOVA-S uses the minimum *P*-values as the test statistic, which is a scale-free approach that is more robust to the various structures of the microbiome data and distances under consideration. We have conducted maxF test on the psoriasis dataset across the six distances and obtained a *P*-value of $1.2\times 10^{-3}$, which is substantially less significant than the *P*-value of our unified test ($3.1\times 10^{-5}$).

The permutation strategy of the unified test proposed in this paper is very general and can be applied to any unified tests minimizing *P*-values. For instance, Zhao *et al.* (2015) cast the problem in the kernel machine framework and developed a regression-based kernel test MiRKAT. Their framework allows for multiple distances. However, the *P*-value calculations of their unified test depend on the asymptotic approximation, which is not accurate when the sample size is modest. The Supplementary Table S3 demonstrates that MiRKAT tends to be overly conservative with small sample size. Our permutation strategy can be readily implemented to improve the small-sample performance of their test.

Failure to take into account the sampling variation can cause inflated beta diversity and produce false positive results. To overcome the potential adverse effects of unbalanced sampling, rarefaction is usually adopted to subsample reads to equal depth. However, if the signal is mainly driven by the change of evenness, throwing away large numbers of observations adversely affects the power. Fortunately, the abundance distances based on the unrarefied data stabilize very quickly with increasing depths (Supplementary Tables S4 and S5). As the read depth is usually large (>1000) in most target sequencing studies, we suggest to use unrarefied data for the abundance distances and rarefied data for the presence–absence distances when testing the association of microbiome compositions. Of note, rarefaction does not always obscure association when the presence–absence distances are used (see power of the $D_{\text{PW}}^{\left(1\right)}$ in Supplementary Tables S4 and S5). For instance, the *P*-value for $D_{\text{PW}}^{\left(1\right)}$ becomes $2.5\times 10^{-4}$ if we use the unrarefied psoriasis data, which shows much weaker evidence of the association than the reported *P*-value using the rarefied data ($1.1\times 10^{-5}$).

Many microbiome studies are being conducted with more complicated designs and heterogeneous samples. We expect that the use of PERMANOVA-S for the accommodation of confounders and multiple distances will enable more robust discoveries in microbiome research.

## Supplementary Material

Supplementary Data
