# Supplementary material for: PERMANOVA-S: association test for microbial community composition that accommodates confounders and multiple distances
Source: Bioinformatics. 2016 May 19;32(17):2618–25. doi: 10.1093/bioinformatics/btw311 (PMC5013911; doi:10.1093/bioinformatics/btw311)
Supplement: Supplementary Data [file supp_32_17_2618__index.html]

PERMANOVA-S: association test for microbial community composition that accommodates confounders and multiple distances — PERMANOVA-S: association test for microbial community composition that accommodates confounders and multiple distances — Supplementary Data 

# PERMANOVA-S: association test for microbial community composition that accommodates confounders and multiple distances

## Supplementary Data

files

- Supplementary Data - zip file
