# Supplementary material for: PERMANOVA-S: association test for microbial community composition that accommodates confounders and multiple distances
Source: Bioinformatics. 2016 May 19;32(17):2618–25. doi: 10.1093/bioinformatics/btw311 (PMC5013911; doi:10.1093/bioinformatics/btw311)
Supplement: Supplementary Data [file supp_btw311_suppl_data.zip › supplr_Cleaned.pdf]

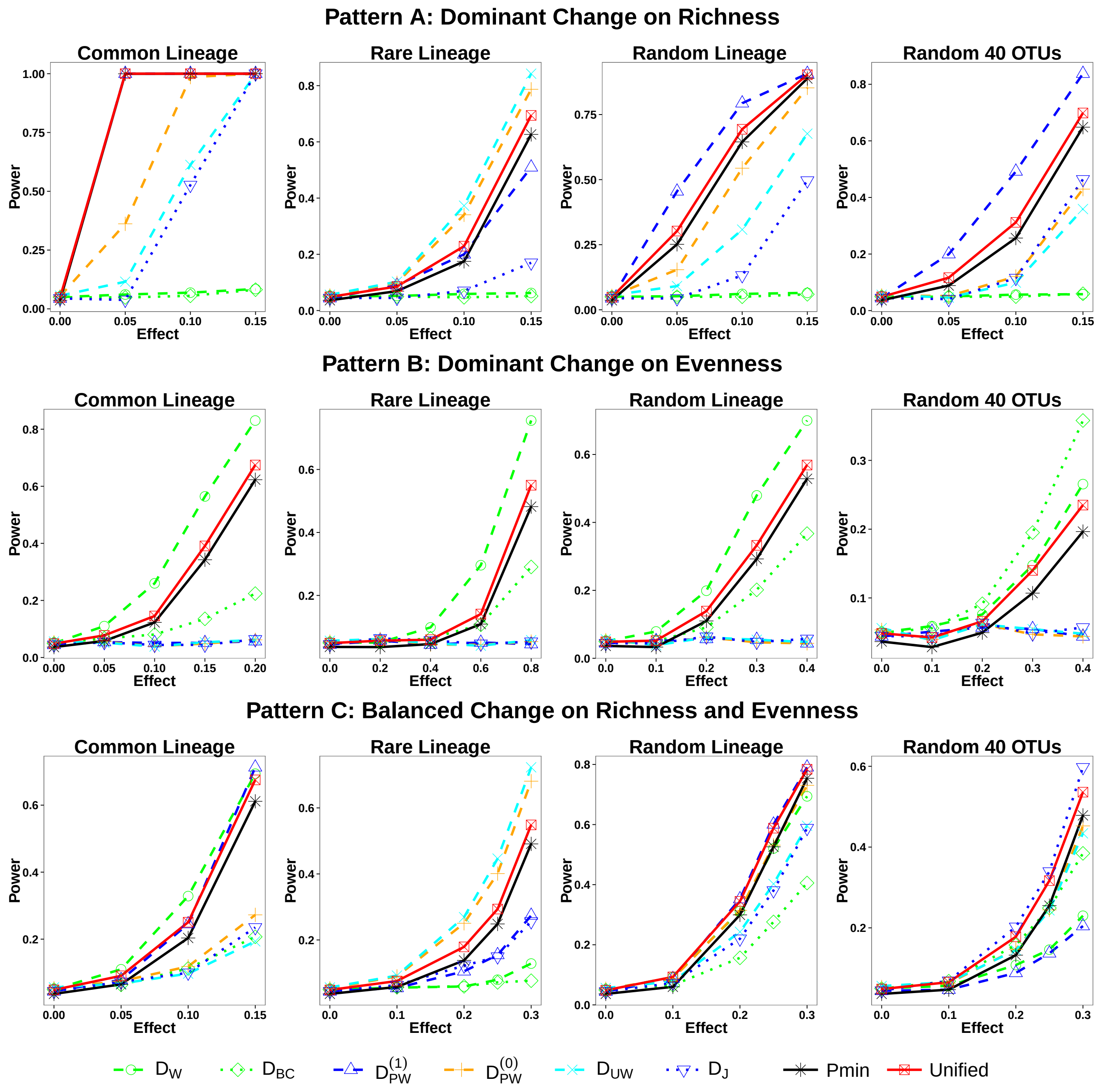

Fig. S1. Power of abundance distances, presence-absence distances, Bonferroni-adjusted test, and PERMANOVA-S unified test under various differentiation patterns. Each curve is created by varying the degree of differentiation between two groups, with 25 samples per group.

Table S1. P-values for detecting differential OTUs between lesion and normal sites of the psoriasis patients. The significant p-values at the level of 0.001 are bold.

OTU	Phylum	Wilcoxon P-value	McNemar P-value
4201	Actinobacteria	$\mathbf{2.5 \times 10^{-4}}$	1.8×10^{-1}
17320	Actinobacteria	6.3×10^{-3}	$\mathbf{5.2 \times 10^{-4}}$
29825	Firmicutes	3.9×10^{-3}	$\mathbf{3.6 \times 10^{-5}}$
15955	Proteobacteria	$\mathbf{1.7 \times 10^{-4}}$	$\mathbf{2.2 \times 10^{-4}}$
27731	Proteobacteria	$\mathbf{5.2 \times 10^{-5}}$	1.3×10^{-2}
6869	Proteobacteria	$\mathbf{1.7 \times 10^{-4}}$	1.8×10^{-1}
18041	Proteobacteria	$\mathbf{1.0 \times 10^{-3}}$	4.2×10^{-3}
25710	Proteobacteria	1.0×10^{-2}	$\mathbf{7.3 \times 10^{-4}}$
23027	Proteobacteria	1.4×10^{-2}	$\mathbf{9.8 \times 10^{-4}}$
2642	Proteobacteria	4.0×10^{-3}	$\mathbf{1.8 \times 10^{-4}}$

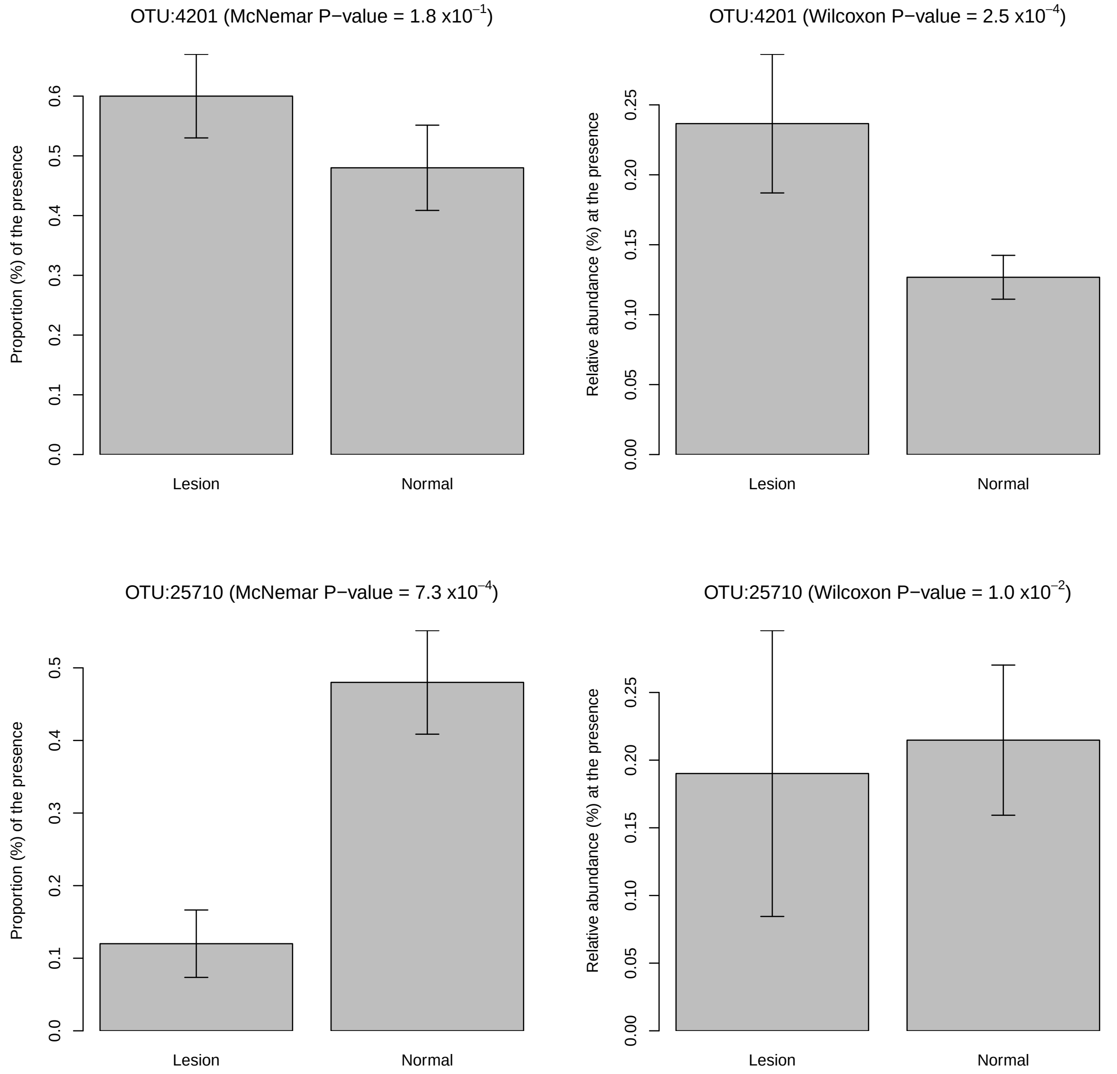

Fig. S2. The proportion of the presences and the mean of the relative abundance at the presences for the lesion and normal groups. The barplots are generated for two OTUs (ID: 4201 and 25710) with distinct results from the Wilcoxon signed rank-sum test and the McNemar's test in Table S1. The p-values are indicated in the graph titles. The confidence intervals for the estimates were shown in the barplots.

Table S2. P-values for testing the association of gut microbiome composition with body fat.

Covariate	Confounder	$\mathbf{D}_W$	$\mathbf{D}_{BC}$	$\mathbf{D}_{PW}^{(1)}$	$\mathbf{D}_{PW}^{(0)}$	$\mathbf{D}_{UW}$	$\mathbf{D}_J$	Unified
mfat[1]	none	0.49	0.17	0.26	0.17	0.30	0.019	0.058
	antibiotics	0.36	0.16	0.27	0.26	0.45	0.071	0.19
pfat[2]	none	0.66	0.29	0.33	0.12	0.25	0.025	0.069
	antibiotics	0.61	0.34	0.35	0.26	0.47	0.16	0.35

[1] mass of body fat [2] percentage of body fat

We performed type I error simulation for MiRKAT. The R package MiRKAT v0.01 is used for the analysis and the simulation strategy is the same as the one in the main text.

Table S3. Type I error for MiRKAT.

n	$\mathbf{D}_W$	$\mathbf{D}_{BC}$	$\mathbf{D}_{PW}^{(1)}$	$\mathbf{D}_{PW}^{(0)}$	$\mathbf{D}_{UW}$	$\mathbf{D}_J$	Unified
No confounder							
20	0.050	0.049	0.049	0.053	0.050	0.051	0.045
50	0.049	0.051	0.048	0.045	0.044	0.050	0.048
1000	0.053	0.050	0.050	0.048	0.049	0.047	0.050
Confounder correlated with abundance							
20	0.040	0.035	0.048	0.030	0.033	0.025	0.036
50	0.049	0.044	0.050	0.042	0.044	0.039	0.037
1000	0.049	0.051	0.052	0.054	0.052	0.050	0.048
Confounder correlated with richness							
20	0.043	0.038	0.044	0.031	0.032	0.027	0.036
50	0.045	0.041	0.047	0.044	0.043	0.041	0.036
1000	0.046	0.047	0.046	0.049	0.050	0.047	0.045

We use negative binomial (NB) distribution to generate read depths for 20 samples in two groups. Four patterns of read-depth variations were simulated: (1) same means, same small variances (coefficient of variation=0.2); (2) same means, same large variances (coefficient of variation=0.5); (3) different means, same variances; (4) same means, different variances. For the power evaluation, we simulated differentiation pattern C on the common lineage with effect size of 0.3. We used 10,000 replicates for the type I error evaluation and 5,000 replicates for the power evaluation at a significance level of 0.05. Table S4 displays results for average depth of 500 and Table S5 displays results for average depth of 1000.

Table S4. Effects of rarefaction on the type I error and power for association testing of microbiome composition with average sequencing depth of 500.

		$\mathbf{D}_W$	$\mathbf{D}_{BC}$	$\mathbf{D}_{PW}^{(1)}$	$\mathbf{D}_{PW}^{(0)}$	$\mathbf{D}_{UW}$	$\mathbf{D}_J$
NB with mean=(500,500), variance=(10000, 10000)							
Type I error	Unrarefied	0.048	0.049	0.051	0.052	0.050	0.050
	Rarefied	0.048	0.050	0.052	0.051	0.053	0.047
Power	Unrarefied	0.92	0.39	0.89	0.51	0.35	0.43
	Rarefied	0.91	0.37	0.91	0.47	0.32	0.38
NB with mean=(500,500), variance=(62500, 62500)							
Type I error	Unrarefied	0.049	0.051	0.051	0.052	0.052	0.051
	Rarefied	0.048	0.049	0.050	0.050	0.050	0.050
Power	Unrarefied	0.91	0.35	0.42	0.45	0.32	0.41
	Rarefied	0.85	0.29	0.79	0.39	0.26	0.28
NB with mean=(300,700), variance=(10000, 10000)							
Type I error	Unrarefied	0.054	0.060	1.00	0.58	0.34	0.27
	Rarefied	0.050	0.050	0.051	0.050	0.050	0.053
Power	Unrarefied	0.91	0.40	1.00	0.88	0.62	0.74
	Rarefied	0.85	0.30	0.82	0.39	0.27	0.29
NB with mean=(500,500), variance=(10000, 62500)							
Type I error	Unrarefied	0.050	0.051	0.096	0.058	0.054	0.052
	Rarefied	0.050	0.051	0.050	0.051	0.051	0.046
Power	Unrarefied	0.91	0.38	0.72	0.53	0.37	0.44
	Rarefied	0.86	0.32	0.83	0.40	0.27	0.32

Table S5. Effects of rarefaction on the type I error and power for association testing of microbiome composition with average sequencing depth of 1000.

		$\mathbf{D}_W$	$\mathbf{D}_{BC}$	$\mathbf{D}_{PW}^{(1)}$	$\mathbf{D}_{PW}^{(0)}$	$\mathbf{D}_{UW}$	$\mathbf{D}_J$
NB with mean=(1000,1000), variance=(40000, 40000)							
Type I error	Unrarefied	0.050	0.049	0.051	0.048	0.048	0.050
	Rarefied	0.052	0.048	0.053	0.050	0.049	0.048
Power	Unrarefied	0.93	0.40	0.96	0.58	0.39	0.53
	Rarefied	0.93	0.40	0.96	0.56	0.37	0.47
NB with mean=(1000,1000), variance=(250000, 250000)							
Type I error	Unrarefied	0.052	0.054	0.052	0.050	0.048	0.047
	Rarefied	0.050	0.050	0.054	0.053	0.052	0.051
Power	Unrarefied	0.93	0.42	0.62	0.54	0.38	0.51
	Rarefied	0.90	0.37	0.90	0.47	0.31	0.37
NB with mean=(600,1400), variance=(40000, 40000)							
Type I error	Unrarefied	0.050	0.053	0.98	0.42	0.24	0.20
	Rarefied	0.047	0.048	0.049	0.048	0.05	0.052
Power	Unrarefied	0.93	0.42	1.00	0.84	0.59	0.77
	Rarefied	0.91	0.38	0.92	0.49	0.33	0.40
NB with mean=(1000,1000), variance=(40000, 250000)							
Type I error	Unrarefied	0.051	0.050	0.080	0.052	0.052	0.051
	Rarefied	0.052	0.051	0.051	0.049	0.050	0.044
Power	Unrarefied	0.93	0.40	0.85	0.59	0.41	0.53
	Rarefied	0.92	0.37	0.91	0.49	0.32	0.40
