# Supplementary material for: PERMANOVA-S: association test for microbial community composition that accommodates confounders and multiple distances
Source: Bioinformatics. 2016 May 19;32(17):2618–25. doi: 10.1093/bioinformatics/btw311 (PMC5013911; doi:10.1093/bioinformatics/btw311)
Supplement: Supplementary Data [file supp_btw311_suppl_data.zip › suppl_Cleaned.zip!/suppl/barCI.pdf]

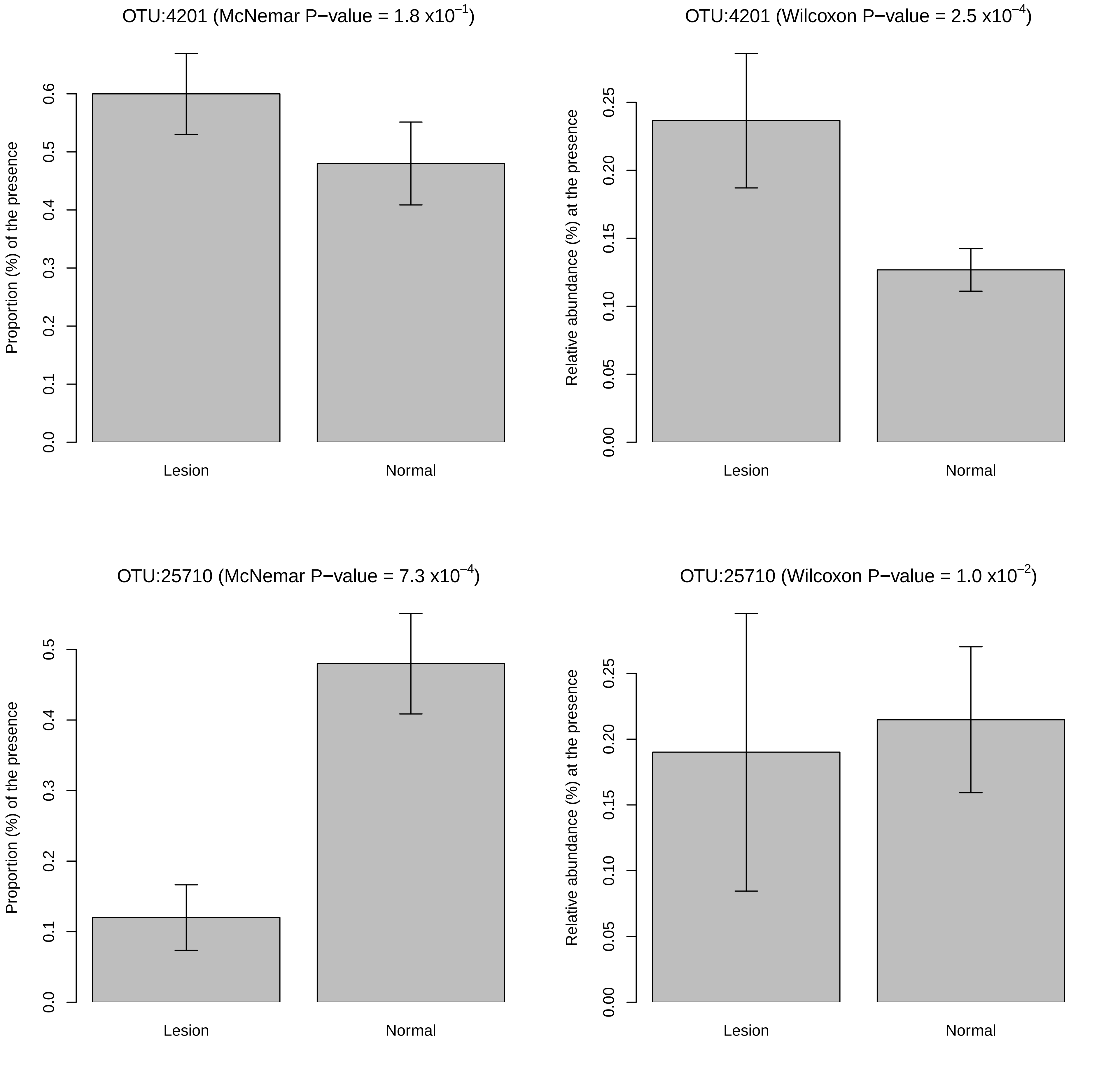

OTU:4201 (McNemar P−value = 1.8 x10−1)
Proportion (%) of the presence
0.0
0.1
0.2
0.3
0.4
0.5
0.6
Lesion
Normal
OTU:4201 (Wilcoxon P−value = 2.5 x10−4)
Relative abundance (%) at the presence
0.00
0.05
0.10
0.15
0.20
0.25
Lesion
Normal
OTU:25710 (McNemar P−value = 7.3 x10−4)
Proportion (%) of the presence
0.0
0.1
0.2
0.3
0.4
0.5
Lesion
Normal
OTU:25710 (Wilcoxon P−value = 1.0 x10−2)
Relative abundance (%) at the presence
0.00
0.05
0.10
0.15
0.20
0.25
Lesion
Normal
