# Supplementary material for: PERMANOVA-S: association test for microbial community composition that accommodates confounders and multiple distances
Source: Bioinformatics. 2016 May 19;32(17):2618–25. doi: 10.1093/bioinformatics/btw311 (PMC5013911; doi:10.1093/bioinformatics/btw311)
Supplement: Supplementary Data [file supp_btw311_suppl_data.zip › suppl_Cleaned.zip!/suppl/fig_sim_power50.pdf]

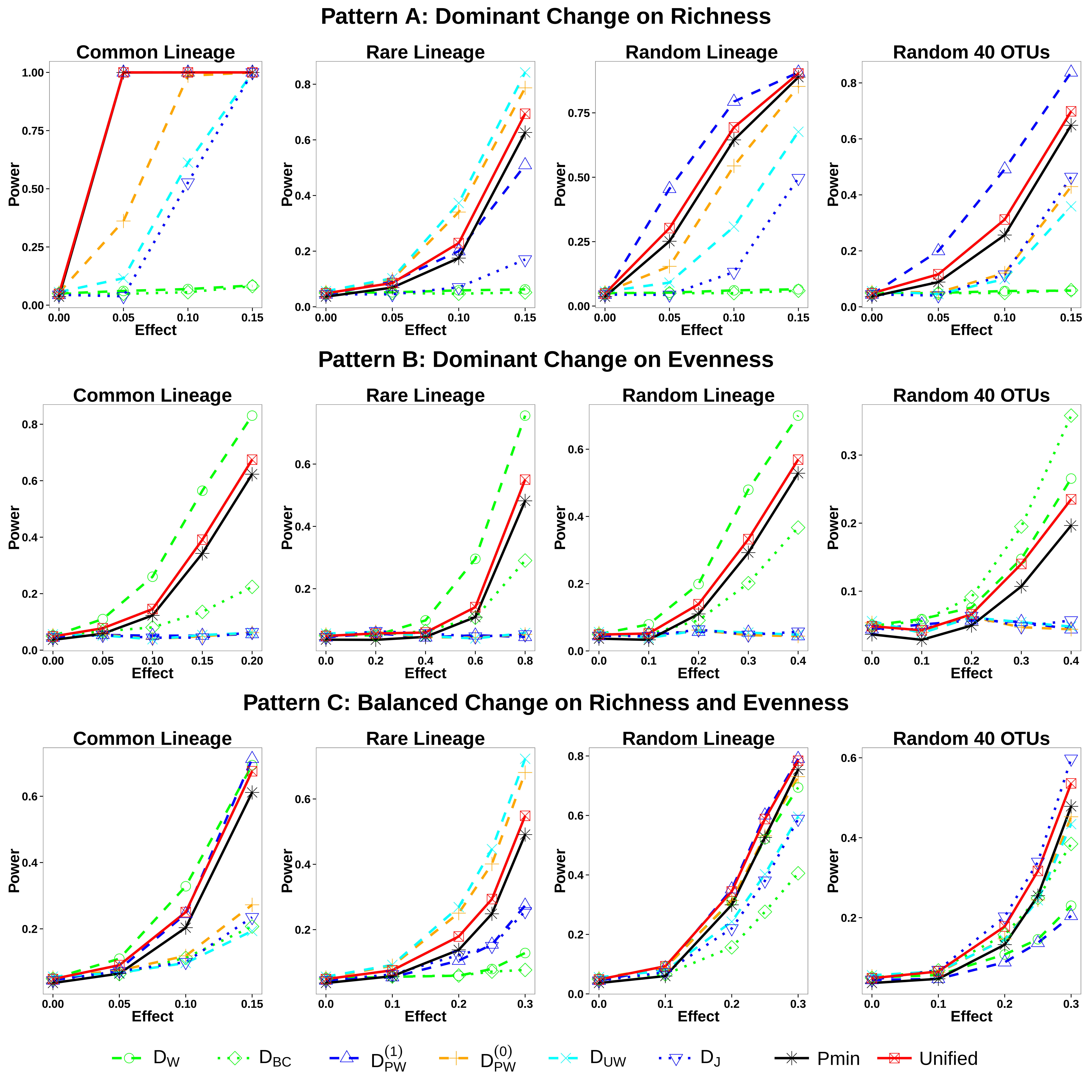

Pattern A: Dominant Change on Richness
Common Lineage
Rare Lineage
Random Lineage
Random 40 OTUs
Pattern B: Dominant Change on Evenness
Pattern C: Balanced Change on Richness and Evenness
Power
Effect
D_W
D_{BC}
$D_{PW}^{(1)}$
$D_{PW}^{(0)}$
D_{UW}
D_J
Pmin
Unified
